# STORMSeq: An Open-Source, User-Friendly Pipeline for Processing Personal Genomics Data in the Cloud

**DOI:** 10.1371/journal.pone.0084860

**Published:** 2014-01-15

**Authors:** Konrad J. Karczewski, Guy Haskin Fernald, Alicia R. Martin, Michael Snyder, Nicholas P. Tatonetti, Joel T. Dudley

**Affiliations:** 1 Biomedical Informatics Training Program, Stanford University School of Medicine, Stanford, California, United States of America; 2 Department of Genetics, Stanford University School of Medicine, Stanford, California, United States of America; 3 Department of Biomedical Informatics, Columbia University, New York, New York, United States of America; 4 Department of Genetics and Genomic Sciences, Mount Sinai School of Medicine, New York, New York, United States of America; Georgia Institute of Technology, United States of America

## Abstract

The increasing public availability of personal complete genome sequencing data has ushered in an era of democratized genomics. However, read mapping and variant calling software is constantly improving and individuals with personal genomic data may prefer to customize and update their variant calls. Here, we describe STORMSeq (Scalable Tools for Open-Source Read Mapping), a graphical interface cloud computing solution that does not require a parallel computing environment or extensive technical experience. This customizable and modular system performs read mapping, read cleaning, and variant calling and annotation. At present, STORMSeq costs approximately $2 and 5–10 hours to process a full exome sequence and $30 and 3–8 days to process a whole genome sequence. We provide this open-access and open-source resource as a user-friendly interface in Amazon EC2.

## Introduction

Individuals are now empowered to obtain and explore their full personal genome and exome sequences owing to declining costs in genome sequencing, and direct-to-consumer genetic testing companies have begun to provide sequencing services: in 2011, 23andMe conducted a pilot exome sequencing program for$999, while at the time of this writing, DNADTC provides the service for $895. Software and algorithms for short read mapping and variant calling are an active area of development and individuals may prefer to customize which software or parameters to use to process their raw genetic data. However, as these programs require significant computational resources, such a task is generally intractable without access to large-scale computing resources. Furthermore, execution of the required software pipeline requires proficiency in command-line programming, or alternatively, expensive commercial software options geared towards experts. These concerns can be ameliorated by use of intuitive open-source software operating in a cloud-computing environment.

A number of solutions enabling researchers to process sequencing data using cloud computing are available. The majority of open-source, cloud-based tools for genomic data are command-line based and require substantial technical skills to use. Notable exceptions are Galaxy, Crossbow, and SIMPLEX. Galaxy aims to provide a reproducible environment for genome informatics accessible to non-technical investigators[1], but offers a vast array of tools beyond those typically used for processing personal genomic data and requires knowledgeable use of its workflow system. Crossbow provides a scalable framework for mapping and variant calling[2], but is limited to the Bowtie suite, while SIMPLEX requires command-line proficiency[3]. Ideally, by our definition, a user-friendly solution would employ a simple, unified graphical user interface for uploading reads, setting parameters, executing analyses, and downloading and visualizing results.

### Implementation

Thus, we created STORMSeq (Scalable Tools for Open-source Read Mapping) to fill the need for a user-friendly processing pipeline for personal human whole genome and exome sequence data. STORMSeq utilizes the Amazon Web Services (http://aws.amazon.com) cloud-computing environment for its implementation, and offers an intuitive interface enabling individuals to perform customized read mapping and variant calling with personal genome data. STORMSeq dissociates the backend computational pipeline from the end-user and provides a simplified point-and-click interface for setting high-level parameters, and the system initiates with an optimized default configuration using recent versions of BWA (0.7.5a) and GATK Lite (2.1) as of 11/1/13. Users can then access final processed data and visualize summary statistics without having to load the data into a statistical software package. STORMSeq is a highly secure system entirely encapsulated within the user's Amazon account space, thereby ensuring that only the user has the ability to gain or grant access to their genetic data and results.

**Figure 1:**
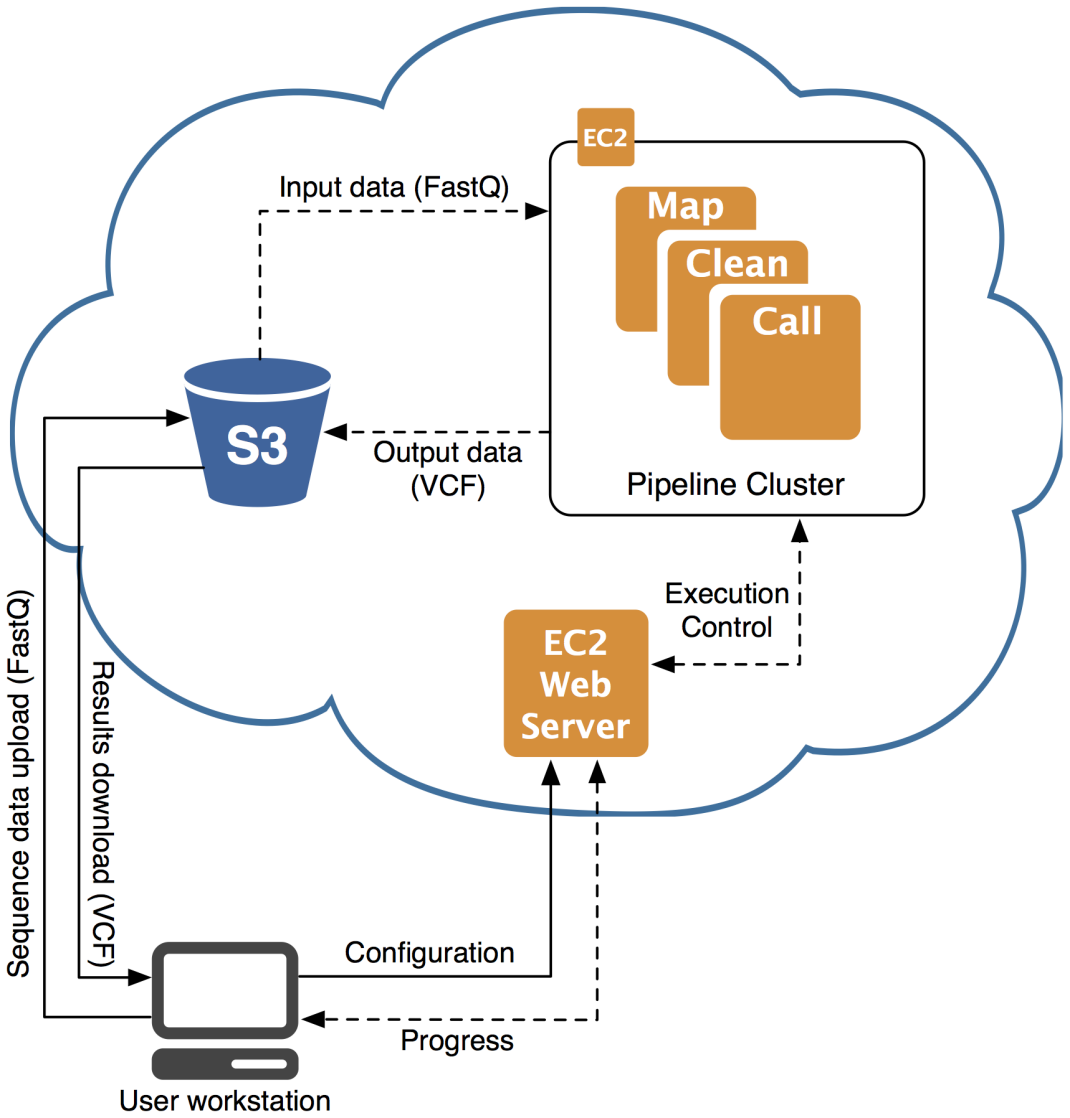
Overview of the STORMSeq system. The user uploads short reads to Amazon S3 and starts a webserver on Amazon EC2, which controls the mapping and variant calling pipeline. Progress can be monitored on the webserver and results are uploaded to persistent storage on Amazon S3.

STORMSeq's cloud-based architecture is illustrated in Figure 1. The user uploads their reads in FASTQ or BAM formats to Amazon S3 (Simple Scalable Storage) through a graphical interface provided by Amazon Web Services. The STORMSeq website (www.stormseq.org) provides instructions for starting the STORMSeq webserver machine image (AMI v1.0: ami-b35b7cda) within the Amazon cloud computing environment. This STORMSeq webserver is then the entry point for the user to choose software packages and set parameters for the analysis (Figure S1). The system currently offers a complete short read processing pipeline, including:

- Read mapping software packages, including BWA[4], BWA-MEM[5], and SNAP[6]
- Read cleaning pipeline with GATK[7]
- Variant (SNP and indel) calling packages, GATK and Samtools[8]
- Annotation using VEP[9]

The system backend is modular, and designed to be easily expandable by researchers wishing to add additional functionality or incorporate other software packages.

**Figure 2:**
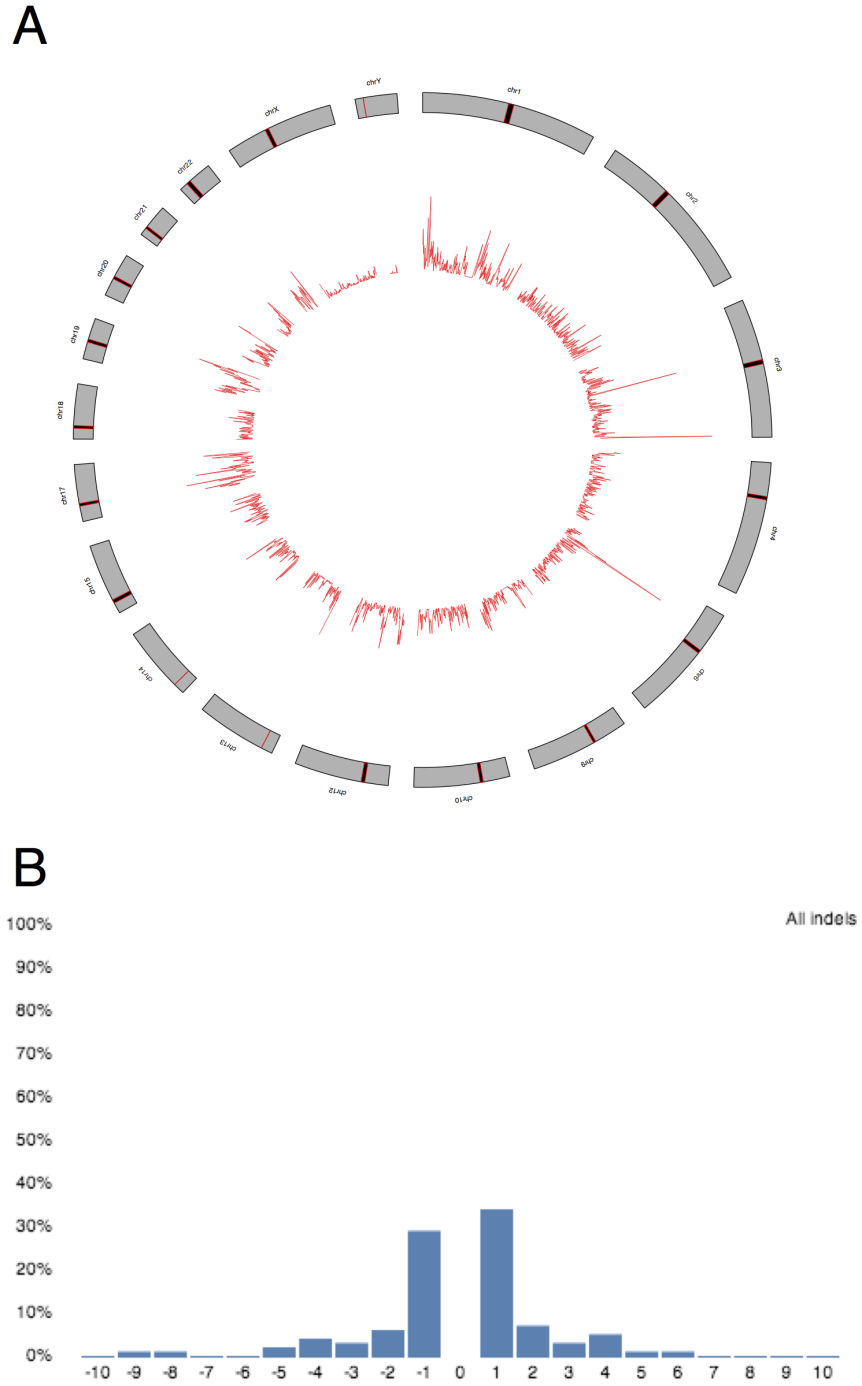
Sample output. STORMSeq provides basic visualization for summary statistics, such as (A) genome-wide SNP density and (B) size distribution of short indels.

Once the user has set the relevant parameters (or uses the default set provided) and clicked “GO,” the system starts a compute cluster on the Amazon Elastic Compute Cloud (with the number of machines started related to the number of files uploaded and whether exome or genome analyses are selected) and runs the relevant software. The use of the software is free, and the user simply pays for compute time and storage on the Amazon servers, which as of 11/1/13 (for spot instances) costs $0.026 per hour for the (large) systems required for BWA, and $0.14 per hour for the (quadruple extra-large) high-memory systems required for SNAP, and $0.095 per GB-month for persistent storage of reads and variant call results. As the pipeline progresses, a progress bar is updated on the webserver and once the pipeline is finished, summary statistics, such as depth of coverage and other variant information, and visualizations using ggbio[10] and d3[11], are displayed on the webserver (Figure 2). Processing is parallelized where possible using Starcluster (http://mit.edu/star/cluster) and Sun Grid Engine. The results are then uploaded back to Amazon S3 for persistent storage.

## Results and Discussion

We tested the STORMSeq system using two paired-end 100 bp read datasets: a personal genome sequence dataset with 1.1B reads (approximately 38X coverage), and a personal exome sequence data set with 90M reads (approximately 45X coverage; available in STORMSeq's demo functionality). For the personal exome data, the pipeline cost approximately $2 USD using spot pricing and took 10 hours using BWA and 5 hours using SNAP (Table 1; Figure S2). For personal genome sequence data, BWA and SNAP took 176 and 82 hours for processing, respectively, and each at a cost of approximately $30 USD (Table 1; Figure S3). Note that these values do not include storage costs, and are highly dependent on a number of factors, including the number and size of files provided by the user, as the software dynamically determines a cluster size based on this information. Additionally, STORMSeq was developed to support current cost savings of spot instances, and so, on-demand costs for the pipeline are much higher (Table 1).

**Table 1:** Approximate costs for STORMSeq.

Analysis Type	Exome		Genome	
Pipeline	SNAP	BWA	SNAP	BWA
Cost (Spot)	$2.26	$1.90	$26.42	$32.76
Cost (On-demand)	$19.68	$8.16	$254.20	$129.12
Time	5 h	10 h	176 h	98 h

Note that these costs are approximate and may depend on a number of factors related to the input files.

We offer STORMSeq free for public use, where users pay only for compute time on the Amazon cloud. The source code for the STORMSeq software is available for download from www.github.com/konradjk/stormseq under an open-source license. We expect that the majority of STORMSeq users will be individuals from academia and the broader lay public interested in analyzing personal genomic information. In addition, those without access to large computing clusters, such as clinicians wishing to process patient data for clinical studies, as well as small research groups with genome sequence projects may seek to use the system to process genomic data for their patients and subjects. The system is modular and can be easily expanded and integrated with other tools. In the future, it will be crucial to integrate such tools with genome interpretation services, such as Interpretome[12].

## Supporting Information

Figure S1 **The STORMSeq webserver allows users to set parameters and start the pipeline using a graphical interface.**
(PDF)

Figure S2 **Time and cost estimates (spot pricing) for a personal exome sequence (90M reads, or 45X coverage) for BWA (red) and SNAP (blue).** These figures are estimates only and results may vary. The merged step includes initial aligned BAMs, while final includes cleaned, sorted, and re-calibrated BAMs, as well as annotated variant calls (VCF). The stats step includes GATK's VariantEval and other VCF statistics, and depth is the completed GATK's DepthOfCoverage process.
(PDF)

Figure S3 **Time and cost estimates for a personal genome sequence (1.1B reads, or 38X coverage) for BWA (red) and SNAP (blue).** These figures are estimates only and results may vary. The merged step includes initial aligned BAMs, while final includes cleaned, sorted, and re-calibrated BAMs, as well as annotated variant calls (VCF). The stats step includes GATK's VariantEval and other VCF statistics, and depth is the completed GATK's DepthOfCoverage process.
(PDF)
