# Supplementary material for: STORMSeq: An Open-Source, User-Friendly Pipeline for Processing Personal Genomics Data in the Cloud
Source: PLoS One. 2014 Jan 15;9(1):e84860. doi: 10.1371/journal.pone.0084860 (PMC3893165; doi:10.1371/journal.pone.0084860)
Supplement: Figure S1 — The STORMSeq webserver allows users to set parameters and start the pipeline using a graphical interface. (PDF) [file pone.0084860.s001.pdf]

STORMSeq

Scalable Tools for Open-source Read Mapping

Setup

Load your data

Create an S3 bucket as described on the STORMSeq page and upload your files.

AWS Account Number:

Access Key ID:

Secret Access Key:

Amazon S3 bucket:

Request type: On-Demand | Spot Request

Hourly price of Large instances as of 3/1/13: $0.24

Set your parameters

Data type: Whole Genome Sequence

Number of genomes: One | Multiple

Name your genome:

Genome version: hg19

dbSNP version: dbSNP 135

Alignment: BWA

Cleaning Pipeline: GATK

SNP Calling: GATK

- [x] Indel Calling
- [x] SV Calling (Coming soon!)
- [ ] Output gVCF (Coming soon!)

Advanced settings

Start processing

GO!

Note that by clicking "GO!", you are starting up Amazon instances and volumes that will be charged to your account.

The mapping and variant calling for a whole genome (30X coverage) is estimated at about $30. For a full exome (50X coverage), the cost is estimated at about $2. However, note that these estimates do not include storage (S3) and that processing times (and thus costs) may vary.

Cancel

Progress

Pipeline progress

Page will automatically refresh every 5 minutes. Click here to refresh manually.

Checking progress...

Download

Download your results

Visualize

Visualize your results

Page will automatically refresh when results are available. Click here to refresh manually.

Refreshing results...
