# Supplementary figures and images for: STORMSeq: An Open-Source, User-Friendly Pipeline for Processing Personal Genomics Data in the Cloud
Source: PLoS One. 2014 Jan 15;9(1):e84860. doi: 10.1371/journal.pone.0084860 (PMC3893165; doi:10.1371/journal.pone.0084860)

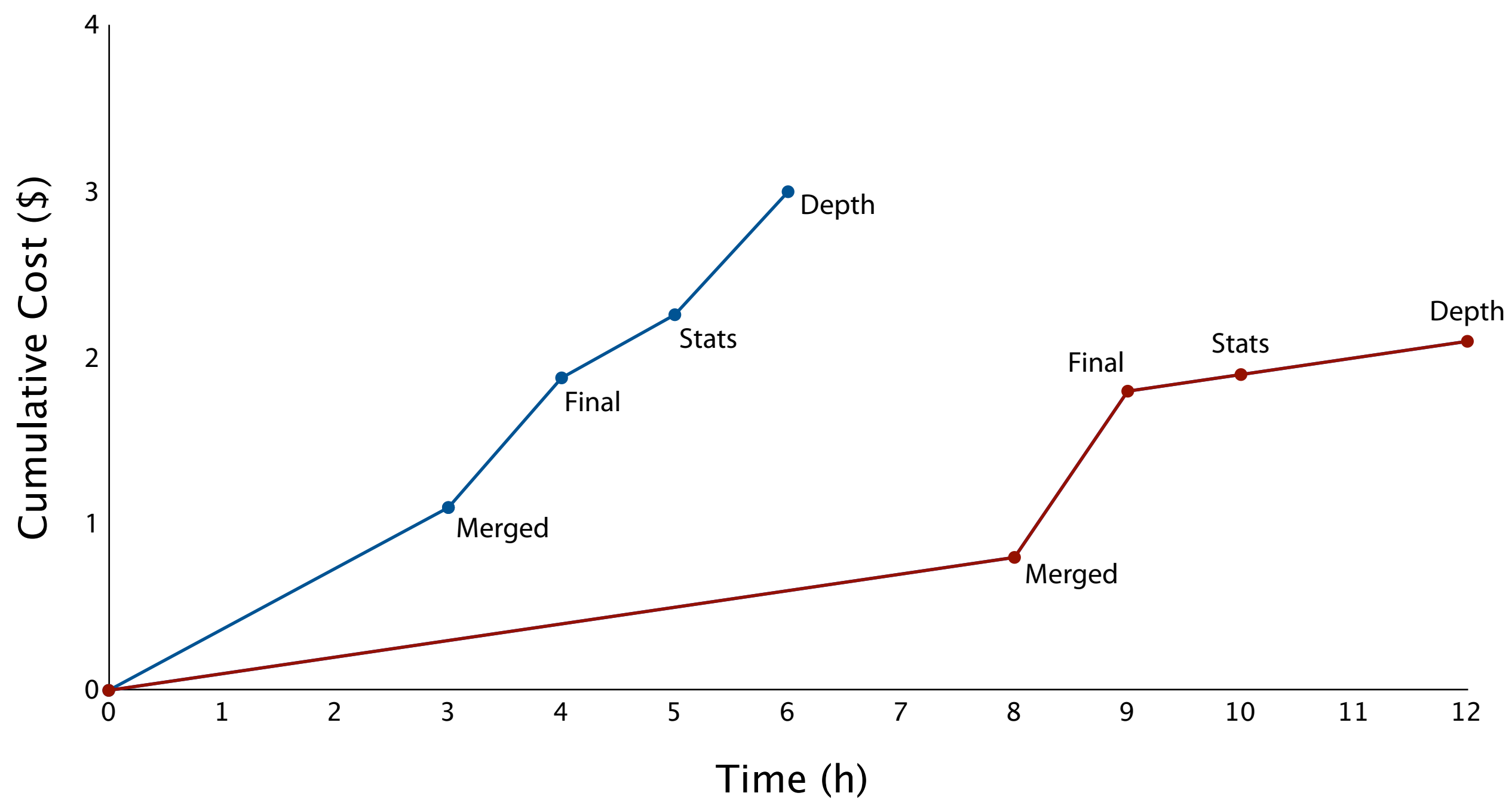

Supplement: Figure S2 — Time and cost estimates (spot pricing) for a personal exome sequence (90M reads, or 45X coverage) for BWA (red) and SNAP (blue). These figures are estimates only and results may vary. The merged step includes initial aligned BAMs, while final includes cleaned, sorted, and re-calibrated BAMs, as well as annotated variant calls (VCF). The stats step includes GATK's VariantEval and other VCF statistics, and depth is the completed GATK's DepthOfCoverage process. (PDF) [file pone.0084860.s002.pdf]

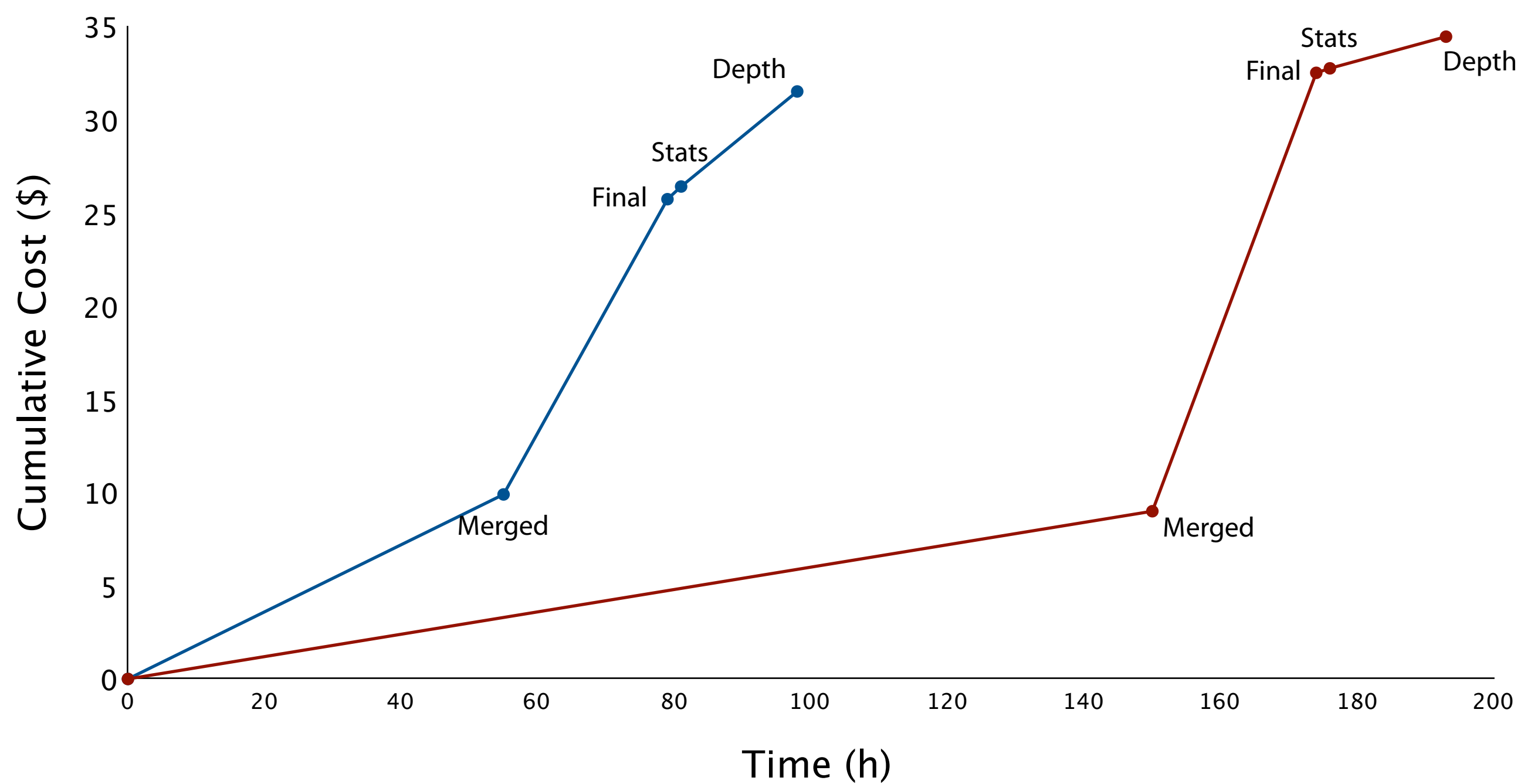

Supplement: Figure S3 — Time and cost estimates for a personal genome sequence (1.1B reads, or 38X coverage) for BWA (red) and SNAP (blue). These figures are estimates only and results may vary. The merged step includes initial aligned BAMs, while final includes cleaned, sorted, and re-calibrated BAMs, as well as annotated variant calls (VCF). The stats step includes GATK's VariantEval and other VCF statistics, and depth is the completed GATK's DepthOfCoverage process. (PDF) [file pone.0084860.s003.pdf]
